# Education Research: Learning to Prognosticate: An Educational Needs Assessment with Senior Child Neurology Residents

**DOI:** 10.1212/NE9.0000000000200306

**Published:** 2026-05-01

**Authors:** Grant L. Lin, Shelby D. Burk, Beatrice D. White, Jessica A. Moriarty, David B. Mauser, Claire E. Wakefield, Courtney J. Wusthoff, Justin Nathaniel Baker

**Affiliations:** 1 Division of Child Neurology, Department of Neurology and Neurological Sciences, Stanford University School of Medicine, CA;; 2 Division of Primary Care and Population Health, Department of Medicine, Stanford University School of Medicine, CA;; 3 Division of Quality of Life and Pediatric Palliative Care, Department of Pediatrics, Stanford University School of Medicine, CA; and; 4 Division of Child Neurology, Department of Neurology, University of California-Davis.

## Abstract

**Background and Objectives:**

Pediatric neuroprognostication is technically challenging and ethically complex. Despite its importance, little is known about how child neurology residents learn to estimate and communicate neurologic prognosis. Current educational interventions focus on communication but lack pediatric-specific focus. This exploratory qualitative study sought to describe how graduating residents conceptualize pediatric neuroprognostication, their educational experiences, and what gaps they identify in learning to prognosticate.

**Methods:**

Semistructured, one-on-one virtual interviews with senior child neurology residents (postgraduate year 5 or 6) were audio-recorded, transcribed, and deidentified. Participants also completed a postinterview survey on exposure to and perceived effectiveness of different learning modalities. Transcripts were analyzed iteratively using a constructivist grounded theory approach with regular consensus meetings. Preliminary themes and subthemes were presented to participants with opportunity for feedback using a standardized set of member checking questions. The findings of the study were mapped to elements of a targeted needs assessment in Kern's six-step model for curriculum development to help inform future educational efforts.

**Results:**

Fifteen residents representing 12 institutions across 10 states participated, representing diverse program sizes and geographic regions. Residents most frequently reported learning neuroprognostication through observation of attending physicians and clinical practice, while coaching/direct mentorship was also felt to be effective but less available. Qualitative analysis yielded 4 themes: conceptualizing content of neuroprognostication (for example, framing around common outcome domains), educational contributors (emphasizing the importance of effective modeling and graded autonomy), perceived efficacy in prognostication (such as relying on structured approaches), and identified gaps in education (including specific topics and modalities). These themes were then mapped to elements of a targeted needs assessment using Kern's six-step approach to curriculum development, highlighting opportunities and gaps in current educational approaches.

**Discussion:**

This qualitative study provides important context for considering strategies to improve resident education on neuroprognostication. The identified gaps highlight needs for structured debriefing and feedback with a particular opportunity for coaching-based strategies, consideration of simulation or roleplay-based modules, and focusing content development on pediatric disease-specific and ethical considerations. Limitations include the need for future work focused on educator perspectives. These findings provide a basis for developing neuroprognostication curricula for child neurology residents.

## Introduction

Prognostication of neurologic outcomes in diseases and injuries managed by child neurologists is a technically and ethically challenging task.^1^ Effective prognostication is an essential part of good patient care,^2^ yet little research has studied how child neurology residents learn this skillset. Understanding how these skills are learned and what gaps are present in existing training is critical for designing educational strategies that help child neurologists develop the skills to effectively estimate and communicate neuroprognostic outcomes.

The challenges in pediatric neuroprognostication are multifold. Often, neuroprognostication is associated with high stakes decisions, such as deciding whether to resuscitate a child if they deteriorate or withdraw life-sustaining therapies in the face of brain injury.^3^ However, there is inherent uncertainty around the recovery potential of a developing brain,^1^ and existing evidence to support estimating or assessing prognosis is limited.^3-5^ Effective prognostication also requires family-centered communication, including understanding a family's values and preferences for receiving prognostic information and decision making.^1,6^ This can be further affected by influences of clinician pessimism (a tendency to predict outcomes that are worse than reality)^7^ or disability bias (where disability is assumed to mean poorer quality of life or prognosis).^5^ These challenges can manifest in poor prognostic communication: despite general guidance that clinicians provide a risk estimate when discussing prognosis,^8^ a study of prognostic language used by clinicians in critical neurologic illness found that most statements were nonprobabilistic (characterizing outcomes without offering qualitative or numeric likelihood), particularly when discussing anticipated cognitive outcomes.^9^

Ongoing work is addressing these challenges in neuroprognostication. For example, the ALIGN (Approach, Learn, Inform, Give support, Next steps) framework^6^ was developed with caregiver input as a family-centered communication framework in neonatal neuroprognostication. The ouR-HOPE (Reflection, Humility, Open-mindedness, Partnership, and Engagement) framework^5^ helps clinicians identify personal biases in neonatal neuroprognostication. A set of patient-centered outcome domains specific to children with severe neurologic impairment was developed through interviews with parents and caregivers.^10^ Others have proposed a stepwise conceptual model to systematically approach assessing neurologic prognosis.^11^ Future research topics include addressing populations with rare diseases, accounting for disease trajectories that change over time, and understanding what contributes to improving shared understanding and concordance amongst families and clinicians.^12^

The American Academy of Neurology includes neuroprognostication as a competency in the 2023 “Neuropalliative Care Curriculum for Neurology Residents.”^13^ Existing curricular modules offer starting points for neuroprognostication education but do not necessarily address challenges specific to child neurology. Recently published interventions in neurology education based on VitalTalk^14^ and the Serious Illness Conversation Guide^15^ are focused on prognostic communication but are targeted at adult neurology and focus solely on communication. Meanwhile, the Education in Palliative and End-of-Life Care Neurology curriculum^16,17^ includes modules on both prognostic communication and disease-specific content but is similarly directed for adult neurology and lacks pediatric-specific disease content. This is particularly challenging in child neurology, where there are gaps in the prognostic data available for specific diagnoses or settings.^4,5^ For instance, a recent study found child neurology residents have variable exposure to and desire-specific training for conversations in fetal and neonatal neurology.^18^

Child neurology residents recognize the importance of learning to prognosticate and express strong desire and motivation for additional education on neuroprognostication.^19^ Here, we conducted qualitative interviews with graduating child neurology residents on their educational experiences with neuroprognostication. This was part of a larger investigation that also explored emotional impacts related to uncertainty in neuroprognostication. This manuscript represents an analysis of (1) how residents conceptualize the content of neuroprognostication, (2) how they describe learning to prognosticate, and (3) what gaps they identify in reflecting on their educational experiences with prognostication. To help inform future educational efforts, we also mapped the findings of this study onto elements of a targeted needs assessment in Kern's six-step approach,^20^ a widely established approach to curriculum development in medical education.

## Methods

### Study Design

This exploratory qualitative study used a constructivist grounded theory approach^21^ to generate an interpretive account of how child neurology residents learn about neuroprognostication. Consistent with constructivism, we assumed residents' experiences reflect multiple, context-dependent realities and treated findings as an interpretive account developed through iterative, inductive analysis. This manuscript adheres to Standards for Reporting Qualitative Research^22^ and Consolidated Criteria for Reporting Qualitative Research^23^ guidelines.

### Research Team and Reflexivity

Interviews were conducted by a male child neurology resident interested in neuropalliative care (G.L.L.) during his postgraduate year 5 (PGY-5). Having a resident interviewer at the same level of training empowered rapport building and allowed in-depth exploration of resident experiences. To mitigate peer-to-peer social desirability bias, participation was voluntary, confidentiality was emphasized, and a thoroughly piloted, semistructured interview guide (see below) was used to promote consistency. The interviewer was trained in medical education research methods through a residency scholarly concentration track and a health professions education certificate program.

The multidisciplinary research team included 2 clinical research coordinators (S.D.B. and B.D.W.), pediatric palliative care attendings with experience in education, qualitative methods, and prognostication (J.A.M., D.B.M., J.N.B.), a mixed-methods research professor with expertise in pediatric serious illness (C.E.W.), and a child neurology attending with background in bioethics (C.J.W.). Team members met regularly to discuss emerging findings, reflect on positionality and assumptions, and reach consensus on coding and thematic development.

### Participant Selection and Recruitment

Participants were child neurology residents in their PGY5/6 years enrolled in ACGME-accredited programs. A convenience sample of graduating residents identified in a prior survey^19^ (n = 19) was recruited through email in August 2024, with additional purposive snowball sampling via direct email to referrals from participants to enhance variation by geographic and program size through March 2025. To avoid role conflicts, residents from the interviewer's home program were excluded. Prior acquaintance with the interviewer (e.g., at conferences), which could affect willingness to participate and social desirability bias, was disclosed to participants before consent, with reiteration that participation was voluntary. Participants provided electronic consent and confirmed review of the research information sheet before interviews. Participants received a nominal $50 cash incentive.

### Sample Size

Sample size was guided by the information power framework^24^ in addition to a goal to achieve thematic saturation.^25^ Given our narrow aim (child neurology resident educational experiences with prognostication), high sample specificity (PGY-5/6 child neurology residents), strong interview quality (piloted guide, trained peer clinician-interviewer, professional transcription), and cross-case thematic analysis, we anticipated 12–18 interviews. Initial coding meetings began after the first 2 interviews to discuss emerging concepts and themes to help focus subsequent interviews. A preliminary codebook was formalized after 11 interviews (described below). We concluded recruitment at 15 interviews when additional interviews contributed minimal novel concepts and cross-case patterns were consistent, indicating thematic saturation and that we had achieved adequate information power.

### Data Collection

#### Demographics and Educational Experiences Survey

Participants completed a Qualtrics survey (eAppendix 1) with items designed to characterize the sample and capture participants' self-reported exposure and perceived adequacy of training. As the survey was meant to provide context for qualitative interviews and to minimize respondent burden and support completion, the survey was kept brief. Items were reviewed by the research team and piloted with recent child neurology resident graduates to assess for clarity and face validity.

#### Qualitative Interviews

A semistructured interview guide (eAppendix 2) including questions based around conceptual models of moral distress^26^ was developed through iterative research team meetings. Pilot interviews were conducted with 4 recent child neurology residency graduates (within 2 years of completing training), which informed additional modifications including the provision of example prompts for different neuroprognostication scenarios and the addition of a working definition of neuroprognostication (“the process of using available information to estimate the expected course of a neurologic disease or injury, likelihood of a cure, functional outcomes, and life expectancy”). The final guide included 11 questions, including 1 question about conceptualization of neuroprognostication and 2 questions on how residents learned and wish they learned about prognostication. The updated guide was used for one-on-one Zoom interviews that were audio-recorded and professionally transcribed using a HIPAA-compliant workflow (TranscribeMe! San Francisco, CA). As we used a single interviewer, field notes were not systematically collected as part of the study protocol to help maintain rapport and interview flow. No repeat interviews were conducted. Transcripts were not returned to participants.

### Data Analysis

Demographic survey data were summarized in Microsoft Excel (Redmond, WA) using descriptive statistics and data visualization.

Deidentified transcripts were managed in Dedoose (Sociocultural Research Consultants, LLC, Los Angeles, CA) to support team-based coding. Consistent with constructivist grounded theory, analysis proceeded iteratively and concurrently with data collection. Three analysts (G.L.L., S.D.B., B.E.W.) read the first 11 transcripts for familiarization and conducted initial line-by-line coding on a subset of transcripts (n = 4) to develop focused themes. The team met regularly to create a working codebook, with an audit trail of coding decisions and category revisions.

To enhance dependability, shared understanding, and minimize coder drift, the 3 analysts reapplied the working codebook to a subset of transcripts (n = 6, including the previously coded plus 2 new transcripts), discussed discrepancies, and further clarified definitions and boundaries. This also followed common guidance that at least 10% of transcripts are group coded to enhance analytic rigor.^27,28^ The lead author (G.L.L.) coded the remaining transcripts (n = 9), with memoing and team meetings to review emergent themes, disconfirming cases, and conceptual sufficiency.

Final qualitative analysis synthesized themes and their relationships to construct an interpretive account of how graduating child neurology residents conceptualize, learn, and practice neuroprognostication. Themes and preliminary interpretations were shared with participants who opted in to provide anonymous feedback using standardized member checking questions.^29^

Given this manuscript's focus on residents' educational experiences, final themes and data were mapped to the elements of a targeted needs assessment in Kern's six-step approach,^20^ a widely established approach to curriculum development in medical education. The second step, a targeted needs assessment, focuses on content including 7 topics about the targeted learners and 5 topics about the targeted learning environment.

### Standard Protocol Approvals, Registrations, and Patient Consents

The study protocol was reviewed and approved by the Institutional Review Board at Stanford University (IRB protocol #75477). Informed consent was obtained from all participants. Deidentified transcripts were stored on secure servers with restricted access.

### Data Availability

Anonymized data not published within this article will be made available by request from any qualified investigator.

## Results

### Demographics and Learning Modalities

A total of 15 residents participated in interviews (Table 1). Interviews lasted a mean of 46 minutes (range 31–61). Of the 19 residents identified in a previous study, 7 (37%) participated; additional participants were recruited through purposive snowballing. The mean age was 32.5 ± 2.7 years, with 11 (73%) identifying as female. Participants represented programs of multiple sizes (average class size 4.4 ± 2.1, range 2–8) with representation across the US (12 programs across 10 states). Of the participants, 10/15 (67%) agreed that they had received sufficient education regarding estimation of neurologic prognosis (3/15 (20%) disagreed), and 13/15 (87%) agreed that they had received sufficient education regarding communication of uncertainty (none disagreed).

**Table 1:** Participant Demographics and Educational Rating

Participant demographics		
Age (Average [SD])	32.5 (2.7)	
Female, (%)	11	73
Region, (%)		
Northeast	5	33
Midwest	4	27
South	4	27
West	2	13
Class Size		
Range	2–8	
Average (SD)	4.4 (2.1)	
I have received sufficient education regarding, (%)		
Estimating neurologic prognosis		
Strongly agree	4	27
Slightly agree	6	40
Neither agree nor disagree	2	13
Slightly disagree	3	20
Strongly disagree	0	0
Communication of uncertainty		
Strongly agree	3	20
Slightly agree	10	67
Neither agree nor disagree	2	13
Slightly disagree	0	0
Strongly disagree	0	0

Participants were asked to report what learning modalities they had received for education on estimating prognosis and communicating uncertainty and then asked to rank the learning modalities based on perceived effectiveness for education on these topics (Figure). The top 3 modalities by perceived effectiveness were observation of attendings, clinical practice, and coaching/direct mentorship. Of these, participants more often reported exposure to observation of attendings (estimating prognosis 100%, communicating uncertainty 93%) and clinical practice (estimating prognosis 100%, communicating uncertainty 87%), with relatively less exposure to coaching/direct mentorship (estimating prognosis 33%, communicating uncertainty 27%).

**Figure:**
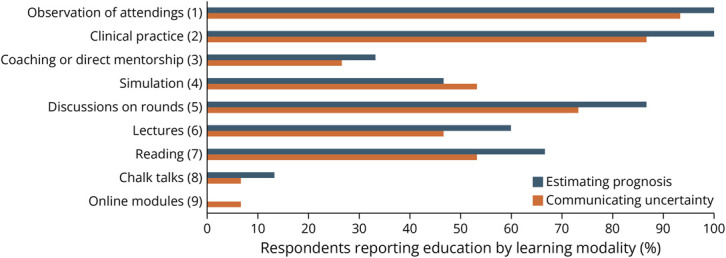
Reported Learning Modalities by Perceived Effectiveness Percentage of respondents reporting receiving education on estimating prognosis (blue) or communicating uncertainty (orange) by each learning modality. Numbers in parentheses represent average rank for perceived effectiveness for teaching these topics.

### Educational Experiences With Prognostication

Qualitative analysis led to 4 themes related to how child neurology residents learn to prognosticate: (1) how they *conceptualize the content of neuroprognostication*, (2) the *educational contributors* to their learning, (3) their *perceived efficacy in neuroprognostication*, and (4) *identified gaps in education* on neuroprognostication. Major themes and subthemes are summarized in Table 2.

**Table 2:** Overall Themes and Subthemes

Overall themes	Subthemes
Conceptualizing content of neuroprognostication	• Calibrating prognostication to medical circumstances
	• Using medical inputs/data
	• Framing around common outcome domains
	• Discussing disease trajectory and rehabilitation
Educational contributors	• Importance of effective modeling
	• Opportunity for graded autonomy
	• Learning from long-term outcomes
	• Drawing upon nonclinical experiences
Perceived efficacy in prognostication	• Using structured approaches
	• Sense of competence
	• Worries about competence
Identified gaps in education	• General need for child neurology-specific curriculum
	• Limitations in current training
	• Opportunities for mentorship, debriefing, feedback
	• Specific topics or modalities

### Theme 1: Conceptualizing Content of Neuroprognostication

Residents shared several factors that contribute to how they conceptualize the content of neuroprognostication (eTable 1). For instance, residents reported *calibrating prognostication to medical circumstances*: “It depends on the setting and who's asking. There's times where a service is basically asking about futility… there's times when we're [preparing] families for what the future looks like… And then sometimes…it's just families asking for, ‘What does this mean?” [Resident 9]. These circumstances also refer to patient age, population, or disease process: “The fact that different populations have different diseases, it warrants different prognostic discussions” [Resident 2].

Residents also described *using medical inputs and data* in their prognostication. This included considering clinical presentation (“Every patient's story is a little bit different. This patient was down for like 120 minutes. There are some patients who are down for 5 minutes” [Resident 15]), neuroanatomy (“I think about it in terms of area hit…to give some sense of what modalities will be potentially hit” [Resident 2]), and other neurodiagnostics like electroencephalograms (EEG). However, 1 resident noted shortcomings of presenting only data: “I think that in your mind, you know what you mean about how terribly this child is going, but a lot of times, we don't directly say that when we round. We just talk about data and data points of labs and EEGs, and that does not translate in the same way” [Resident 10].

Residents framed prognosis around *common outcome domains*. These included predicting activities of daily living (“I like to think of [functionality] as the basic ADLs: walking, talking, feeding themselves, using the restroom, and then higher-level ADLs such as going to school…” [Resident 4]), anticipating cognitive development (“I think the family really wants to know, ‘Cognitively, how impaired is my child going to be, and will they be able to recognize me, interact with me, and interact with their environment in a meaningful way in the future?” [Resident 10]), and describing possible technology dependence (“Depending on the situation, whether they'll need the feeding tube, trach, challenges with swallowing, speaking…” [Resident 6]). As 1 resident summarized: “Those are the big things that families want to hear too. It's like, “Will my kid walk? Will they talk? Will they be the same?” It's kind of the 3 big buckets that I feel like families want to know about” [Resident 15].

Finally, residents discussed including *disease trajectory and rehabilitation* as part of prognostication. For instance, 1 resident explained how they make a point to talk about disease trajectory “depending on the underlying etiology, risk for recurrence or relapse or exacerbation down the line, because I think that that's oftentimes something that's not routinely discussed in neuroprognostic conversations” [Resident 13]. Another resident noted that they stress the importance of rehabilitation: “If we do not pursue the therapies, then they won't have the opportunity to be as independent as they can be” [Resident 3].

### Theme 2: Educational Contributors

Residents named several contributors to how they learned about neuroprognostication (eTable 2). One common contributor was the *importance of effective modeling*: “Seeing other people model it for me and people who've been practicing for a long time… you try to take away the things that are done well” [Resident 10]. This included examples of setting up the conversation, specific language, and de-briefing. However, there was also the challenge of poor modeling (“I've seen other people leave it at that and say like, “It could be anything.” I think that is frustrating and somewhat of a disservice…” [Resident 14]). The importance of attending physicians who are effective prognosticators is amplified given that residents felt observation of attendings was most the most effective learning modality.

Another contributor was the *opportunity for graded autonomy* to practice delivering prognostic information: “You can watch someone do it a million times, but until you do it, you're not going to know…you have to take those chances yourself to say it, to feel it” [Resident 12]. Residents were appreciative of when attendings supported this desire: “I have some attendings who are…very proactive about making sure that resident voices are heard…I have 1 attending who really tends to ask me kind of what I think and how I felt like an experience went” [Resident 2].

Residents also described *learning from long-term outcomes*, such as when outcomes differed from what they anticipated during initial prognostication: “I discussed the prognosis for a baby I saw in the NICU. And I saw them outpatient…the patient, even though she was delayed, was doing very well, much better than I expected. And I remember I painted a pretty- I don't want to say grim picture, but I was very clear… But she was doing very well” [Resident 6]. One resident shared that “we're really bad at prognosticating because most patients do way better than we think they will in the acute window” [Resident 12], highlighting that these exposures can counteract clinician biases towards pessimism in prognostication.

Finally, a subset of residents described *drawing on nonclinical experiences*. For instance, 1 resident shared that they had completed a separate program in spirituality, theology, and health, another resident spoke about how being a parent changed how they think about prognostication, and a third spoke about having participated in research about prognostic biomarkers before residency.

### Theme 3: Perceived Efficacy in Neuroprognostication

Consistent with the postinterview survey data, most residents described that they were able to effectively neuroprognosticate (eTable 3). Several residents described *using structured approaches* to discussing uncertainty and prognosis. One resident explained that they name uncertainty at the start of prognostic conversations: “I lead with a very regular script in that I don't have a [magic] 8 ball…by starting out with that, I always feel like I set myself up for success…because I'm saying that there's no way I could 100% tell you anything” [Resident 1]. Another shared that they cover outcome domains in a set order as “a way for me to know that I kind of hit everything” [Resident 6].

Residents also shared that their training has given them a *sense of competence*: “As you keep doing it over time, it does build a certain level of confidence. Over the last 3 years or so, I have definitely been able to build kind of an increased level of comfort with having those conversations” [Resident 10]. These repeated experiences expand the skillset for residents: “I think you have more language. You have more tools in the toolbox as you go through it” [Resident 15]. Still, consistent with the postinterview findings that some residents disagreed they had received enough education on estimating prognosis, a subset of residents shared *worries about competence*. For instance, in discussing a situation in which there were disagreements about prognostication amongst medical teams, one resident said, “I don't think I have enough knowledge and training to feel comfortable to speak up and say what I'm thinking” [Resident 2].

### Theme 4: Identified Gaps in Education

Residents expressed a *general need for child neurology-specific curriculum* on neuroprognostication, since “a lot of it is just from learning by seeing and learning by doing. I wish we had better formal education for it” [Resident 6]. They highlighted that “it would be nice if it was kind of tailored more towards neurology because the questions we get asked are different than what some other specialties might get asked” [Resident 10]. Another resident highlighted the importance of building early frameworks, noting “I feel like there might be an assumption that we all know what way things are going to go. But honestly, I think especially young trainees that are just starting out…it would be really helpful to create that frame of reference” [Resident 15].

Residents also named *limitations in current training*. For instance, 1 resident noted the difficulty of learning to prognosticate from studying: “It's really hard to read in a textbook what to expect for a patient…I think just based off of clinical experience, that's kind of what I use at this point in my training to guide my discussions with families” [Resident 2]. Another resident noted that “because the neurology portion of our training is so short, that continuity of care often isn't there to build those long-standing relationships for these really complex neurologically devastated kids” [Resident 8].

Residents described *opportunities for mentorship, debriefing, and feedback* to help with education. One resident shared that “we have excellent faculty who I think have been great mentors for me, but I don't know if everyone's had that experience, honestly” [Resident 12]. Another resident described that it is important to make space for formal debriefs and have trained faculty “making it so that you can actually get feedback…And that takes training. It takes time. It takes people getting feedback on their conversations” [Resident 15].

Finally, residents suggested several *specific topics or modalities* to consider for education on neuroprognostication (eTable 4). This included including diagnosis-specific teaching that could help tailor prognosis especially for rare diseases; understanding the ethical implications of prognostication, such as for situations with patients from different cultures or belief systems; emphasizing the applicability of simulation or roleplay to get hands-on practice; communication skills training; and training on conveying statistical information and uncertainty.

### Mapping to Elements of a Targeted Needs Assessment

To help frame our findings for future curricular development, we mapped our findings to relevant content from Kern's targeted needs assessment.

1. Expectations regarding scope of knowledge and skills needed: Residents conceptualized neuroprognostication as a medical context-dependent opportunity to use medical inputs and data to predict and communicate expectations for common outcome domains and disease trajectory for a given patient.
2. Previous training and experiences: Some residents identified that other professional roles and personal experiences contribute to their approach to neuroprognostication.
3. Already planned training/related existing curricula: Residents report learning about neuroprognostication predominantly through modeling in the clinical environment and opportunities for graded autonomy of practice.
4. Existing characteristics/proficiencies/practices: Residents described applying structured approaches to communication and relying upon repeated practice to develop additional tricks and techniques.
5. Perceived learning needs: Residents named several specific topics as gaps in learning, as well as the need for structured debriefing and feedback in clinical settings and the opportunity for simulation or roleplay-based modules.
6. Attitudes and motivations of learners: Residents described a general need for child neurology–specific curriculum on neuroprognostication.
7. Preferences and experiences regarding different learning strategies: We identified mismatch in the perceived effectiveness of and exposure to coaching and direct mentorship as an educational strategy.
8. Informal and collateral curricula: One example of an informal curriculum is how residents adjust their prognostication by learning from long-term outcomes.

## Discussion

This qualitative study describes how child neurology residents conceptualize and learn about neuroprognostication. Residents conceptualize neuroprognostication as applying medical inputs to anticipate trajectories and core functional outcomes, and they largely learn by watching faculty attendings and practicing at bedside. They describe using pragmatic tools, such as scripts for naming uncertainty and domain-based outcome framing but also identify unmet needs in disease-specific knowledge, ethical considerations, and practice with feedback. While many reported a sense of competence, confidence varied and may reflect limitations in mentorship and feedback; moreover, confidence may not represent actual competence in effective neuroprognostication. Taken together, these findings highlight an opportunity to move from opportunistic exposure toward more structured curriculum that integrates coaching, simulation, and longitudinal follow-up. The reliance on effective modeling also highlights the need to strengthen faculty development to ensure consistent, high-quality modeling.

Applied as a targeted needs assessment in Kern's 6-step approach to curriculum development,^20^ our findings provide context for considering strategies to improve resident education on neuroprognostication. For instance, mapping how residents conceptualize neuroprognostication to existing frameworks of neuroprognostication^11,30,31^ can highlight components to target in prognostication curricula. Actively identifying and naming aspects of their lived experience that may alter their approach to neuroprognostication—such as through guided reflection^32^—can help foster a humanistic connection to clinical work in challenging situations. Reflecting on how personal experience informs prognosis is also a debiasing step in the ouR-HOPE framework for ethically informed neuroprognostication.^5^ Systematically incorporating exposure to long-term outcomes, such as in longitudinal clinics or structured follow-up mechanisms, as an intentional educational strategy could represent an underutilized opportunity for education on neuroprognostication.

Notably, our data did not map to some factors of a targeted needs assessment from Kern's model, including measuring deficiencies in knowledge. For instance, the reliance on structured approaches may reflect a need to emphasize curricula that develop flexibility in discussing prognosis, including adapting to patient/family preferences for receiving information and focusing on developing skills around responding to patient/family reactions to receiving prognostic information.^33^ Despite the emphasis on modeling and graduated autonomy, in a previous study,^19^ we found 6% of PGY5/6 child neurology residents reported not having had direct clinical experience with prognostication. Another concern is that the emphasis on modeling by attending faculty who themselves have not had structured curricula on prognostication may perpetuate nonideal practices, such as providing nonprobabilistic prognoses,^9^ contributing to the “self-fulfilling prophecy” (where pessimistic prognostication changes medical decisions and contribute to adverse outcomes),^34^ or using linguistic shortcuts that embed implicit value judgements (e.g., “poor prognosis”).^35^ Recognition of deficiencies in existing prognostic practices highlight the need for caregiver-informed frameworks for prognostic disclosure (e.g., the ALIGN framework),^6^ but ongoing work is required for the implementation, evaluation, and teaching of these practices. Interestingly, during the pilot interview phase, 1 participant described that their approach to neuroprognostication had evolved as an attending to deemphasize functional outcome domains; this did not arise in our interviews with current residents and raises questions if a similar exploration with attending faculty would identify other gaps in neuroprognostication.

This work underscores the need for structured curricula on pediatric neuroprognostication and suggests important next steps. Future didactic or module-based interventions could use case-based approaches to target specific challenges (e.g., rare diseases with limited prognostic data) or frameworks (e.g., ALIGN, ouR-HOPE) in child neurology. Developing a coaching guide or checklist could support direct feedback on specific skills in the clinical setting. Finally, understanding how faculty attendings explicitly or implicitly approach modeling neuroprognostication for residents could inform needs for future faculty development and CME efforts.

This study has several limitations. As a qualitative, constructivist study, we emphasized exploring individual resident experiences with neuroprognostication in-depth, and necessarily did not attempt to assess quantitative estimates of educational exposures across all residents and programs. Moreover, this study was not designed as a formal educational needs assessment, meaning important perspectives such as those of educators were not studied. While we achieved our desired information power and concluded recruitment only after new interviews contributed minimal new concepts, it is possible that additional insights or ideas could be identified with additional participants. We also focused on senior child neurology residents before their graduation and cannot answer the question of how they may feel about their efficacy after completing residency and entering independent practice.

In conclusion, we identify several specific needs and opportunities for the development of neuroprognostication curricula for child neurology residents. The findings from our study can be used as a basis for such a curriculum, with particular emphasis on considering strategies that would address the desire for and gap in exposure to coaching or mentorship based educational models. In addition, our findings underscore the importance of ongoing attending faculty development to promote effective modeling, debriefing, and feedback in the clinical setting.

## Glossary

PGY-5: postgraduate year 5
